# Timing of multikinase inhibitor initiation in differentiated thyroid cancer

**DOI:** 10.1530/ERC-17-0016

**Published:** 2017-03-07

**Authors:** Marcia S Brose, Johannes Smit, Chia-Chi Lin, Fabian Pitoia, Marc Fellous, Yoriko DeSanctis, Martin Schlumberger, Masayuki Tori, Iwao Sugitani

**Affiliations:** 1 Department of Otorhinolaryngology: Head and Neck SurgeryAbramson Cancer Center of the University of Pennsylvania, Stellar-Chance Laboratories Mezzanine, Philadelphia, Pennsylvania, USA; 2 Department of Internal MedicineRadboud University Nijmegen Medical Center, 463 General Internal Medicine, Nijmegen, Netherlands; 3 Department of OncologyNational Taiwan University Hospital, Taipei, Taiwan; 4 Division of EndocrinologyHospital de Clínicas, Universidad de Buenos Aires, Buenos Aires, Argentina; 5 Bayer HealthCare PharmaceuticalsWhippany, New Jersey, USA; 6 Department of Nuclear Medicine and Endocrine OncologyInstitut Gustave Roussy and Université Paris Saclay, Villejuif, France; 7 Department of Endocrine SurgeryOsaka Police Hospital, Tennoujiku, Osaka, Japan; 8 Department of Endocrine SurgeryNippon Medical School Graduate School of Medicine, Bunkyo-Ku, Tokyo, Japan

**Keywords:** differentiated thyroid cancer, multikinase inhibitor, noninterventional, observational, radioactive iodine refractory, sorafenib

## Abstract

There are limited treatment options for patients with radioactive iodine refractory, progressive differentiated thyroid cancer. Although there is consensus that multikinase inhibitor therapy should be considered in patients with progressive disease with considerable tumor load or symptomatic disease, uncertainty exists on the optimal timing to treat with a multikinase inhibitor, especially for asymptomatic patients. RIFTOS MKI is an international, prospective, open-label, multicenter, noninterventional study with the primary objective to compare the time to symptomatic progression from study entry in asymptomatic patients with radioactive iodine refractory, progressive differentiated thyroid cancer for whom there is a decision to initiate multikinase inhibitors at study entry (cohort 1) with those for whom there is a decision to not initiate multikinase inhibitors at study entry (cohort 2). Secondary endpoints are overall survival and progression-free survival, which will be compared between cohorts 1 and 2. Additional secondary endpoints are postprogression survival from time of symptomatic progression, duration of and response to each systemic treatment regimen and dosing of sorafenib throughout the treatment period. Asymptomatic, multikinase inhibitor-naive patients aged ≥18 years with histologically/cytologically documented differentiated thyroid cancer that is radioactive iodine refractory are eligible. Patients may receive any therapy for differentiated thyroid cancer, including sorafenib or other multikinase inhibitors if indicated and decided on by the treating physician. In total, 700 patients are estimated to be enrolled from >20 countries. Final analysis will be performed once the last enrolled patient has been followed up with for 24 months (ClinicalTrials.gov identifier: Nbib2303444).

## Introduction

The majority of thyroid cancers are differentiated thyroid cancers (DTC) developing from thyroid follicular cells and are classified as papillary or follicular carcinomas (Cooper *et al.* 2006, Burns & Zeiger 2010). DTC is associated with a good prognosis; in the United States, 5-year survival of patients diagnosed in 2007 was 97.9% based on data from the Surveillance, Epidemiology, and End Results (SEER) program (SEER 2015). Conventional treatment for thyroid carcinoma involves thyroidectomy followed by remnant ablation with radioactive iodine (RAI) and thyroid-stimulating hormone suppression therapy and is potentially curative (Cooper *et al.* 2006, Bilimoria *et al.* 2007). Postoperative treatment with RAI is recommended for patients with a significant risk of recurrence based on the combination of individual clinical factors, such as the size of the primary tumor, histology, degree of lymphovascular invasion and lymph node metastases, extension beyond the thyroid capsule and postoperative level of thyroglobulin (National Comprehensive Cancer Network 2015). The modified American Thyroid Association (ATA) risk stratification system for predicting the risk of disease recurrence or persistence is a three-tiered continuum of risk that classifies patients as low, intermediate or high risk based on clinicopathological features of the disease as well as the extent of lymph node involvement, mutational status and specific follicular thyroid carcinoma (FTC) histologies (Haugen *et al.* 2016). The prognosis remains favorable even in patients who experience astructural incomplete response. However, once tumors become unresponsive to RAI or unamenable to resection, limited therapeutic options are available, and survival declines rapidly (Durante *et al.* 2006). For example, 10-year survival rate of patients with distant metastases from DTC who achieved negative results on imaging studies after treatment with RAI was 92% compared with 19% in those who did not (Durante *et al.* 2006). Traditional systemic chemotherapies have demonstrated poor efficacy in RAI refractory DTC (Sherman 2011).

Recent advances in understanding of oncogenic pathways of thyroid tumors have enabled the development of targeted therapies for metastatic thyroid carcinoma. Mutations in the mitogen-activated protein kinase signaling pathway have been discovered in about 70% of FTC cases (Santarpia *et al.* 2010). These include the receptor tyrosine kinase RET as well as the signaling molecules RAS and BRAF (Kimura *et al.* 2003, Ho & Sherman 2011). The Cancer Genome Atlas Network analysis of papillary thyroid tumors, the most common thyroid malignancy, found that the majority of tumors had activating mutations in the *BRAF* and *RAS* family genes as well as fusion oncoproteins involving receptor tyrosine kinases (Cancer Genome Atlas Research Network 2014). In the less common poorly differentiated and anaplastic thyroid cancers, mutations in the *TERT* promoter, known to activate its transcription, increased in frequency with more advanced disease and were associated with *BRAF* and *RAS* mutations (Landa *et al.* 2016). Thyroid malignancies are highly vascular tumors; therefore, angiogenesis inhibitors are attractive clinical targets (Ho & Sherman 2011).

Sorafenib is a multikinase inhibitor (MKI) that targets both tumorigenic and angiogenic molecules, including RET, vascular endothelial growth factor receptors 1–3, Flt3, c-KIT and BRAF (Wilhelm *et al.* 2008). It has been approved for the treatment of advanced renal cell carcinoma, hepatocellular carcinoma and progressive RAI refractory DTC. The efficacy and safety of sorafenib in RAI refractory DTC have been established in a multicenter, phase 3 DECISION trial involving 417 patients (Brose *et al.* 2014). Sorafenib treatment significantly improved median progression-free survival (PFS) compared with that in placebo (hazard ratio (HR), 0.59; 95% CI, 0.45–0.76; *P* < 0.001; median 10.8 vs 5.8 months, respectively). The most common treatment-emergent adverse events (TEAEs) were hand-foot skin reaction (76.3%), diarrhea (68.6%), alopecia (67.1%) and rash/desquamation (50.2%) (Brose *et al.* 2014). In a *post hoc* analysis of the DECISION trial, PFS benefit with sorafenib vs placebo was observed both for patients symptomatic (10.7 vs 3.6 months) and asymptomatic (10.8 vs 7.2 months) at baseline (Schlumberger *et al.* 2014).

Lenvatinib is another MKI that was recently approved for the treatment of progressive RAI refractory DTC. In the phase 3 SELECT trial involving 392 patients, PFS was 18.3 months in the lenvatinib group compared with 3.6 months in the placebo group (HR, 0.21; 99% CI, 0.14–0.31; *P* < 0.001). The most common TEAEs were hypertension (67.8%), diarrhea (59.4%), fatigue or asthenia (59.0%) and decreased appetite (50.2%) (Schlumberger *et al.* 2015).

The National Comprehensive Cancer Network guidelines recommend the use of sorafenib and lenvatinib for progressive and/or symptomatic disease (National Comprehensive Cancer Network 2015). However, globally, there is no consensus about when patients with RAI refractory DTC should start MKIs when they are asymptomatic and progressing, and such information, supported by broader evidence-based and long-term outcome data, is needed to guide treatment decisions (Carhill *et al.* 2015).

Radioactive Iodine reFractory Asymptomatic Patients in Differentiated Thyroid Cancer – an Observational Study to Assess the Use of Multikinase Inhibitors (RIFTOS MKI) is a noninterventional study designed to assess the use of MKIs in asymptomatic patients with RAI refractory progressive DTC. The study also aims to evaluate the practice patterns of treating physicians. This article describes the details of the RIFTOS MKI study design, data collection and planned analysis, along with the advantages and limitations of the study.

## Materials and methods

### Study design

This international, prospective, open-label, multicenter, noninterventional study is designed to compare time to symptomatic progression (TTSP) from study entry in asymptomatic patients with RAI refractory, progressive DTC for whom there is a decision to initiate MKIs at study entry (cohort 1) with that of asymptomatic patients with RAI refractory progressive DTC for whom there is a decision to not initiate MKIs at study entry (cohort 2). A propensity score model will be used to balance baseline variables to reduce confounding associated nonrandomized studies. The variables to be taken into account for this model will be defined by the steering committee of the study. This study will also examine the overall survival (OS) and progression-free survival (PFS). Data regarding safety, efficacy and dosing of sorafenib in patients with RAI refractory DTC in a real-world, routine clinical setting will be collected. In addition, practice patterns of physicians involved in the care of patients with RAI refractory DTC will be evaluated.

This trial is being conducted by Bayer HealthCare Pharmaceuticals in >20 countries and is registered at www.ClinicalTrials.gov (Nbib2303444). The study is being carried out in accordance with the guidelines and regulations of the US Food and Drug Administration, the European Medicines Agency and applicable local laws and regulations. Approval from appropriate independent ethics committees or institutional review boards has been obtained, where required, for all participating centers before start of the study. Country and site selection for the study will consider a regional spread to avoid geographic bias, and the number of patients per site will be limited to avoid site bias.

### Patients

#### Inclusion criteria

Asymptomatic, MKI-naive men and women aged 18 years and older diagnosed with locally recurrent or metastatic, progressive, histologically/cytologically documented RAI refractory DTC (papillary, follicular, Hürthle cell or poorly differentiated carcinoma) will be enrolled in a consecutive manner. Patients must have documented radiologic progression preferably according to Response Evaluation Criteria in Solid Tumors (RECIST) 1.1 and ≥1 radiologically confirmed lesion ≥1 cm with the time from radiographic documentation of progression to the initial study visit expected to be ≤3 months. The definition of asymptomatic DTC includes the absence of symptoms before enrollment in the study due to bone metastasis such as bone pain; pathologic fracture due to metastatic lesion; respiratory symptoms such as postobstructive pneumonia, dyspnea and hemoptysis; cough due to metastatic lesion; central nervous system event due to metastatic lesion; bleeding event; local discomfort or pain (such as feeling of pressure in lymph nodes in the neck; liver metastases; or pain in soft-tissue metastases due to metastatic lesion); deterioration of general condition such as worsened Eastern Cooperative Oncology Group (ECOG) perfor­mance status, fatigue and cachexia; reduced mobility due to metastatic lesion or any other symptoms due to DTC. Additionally, patients are required to have life expectancy of ≥6 months and sign an informed consent form before participating.

#### Exclusion criteria

Patients will be excluded if they plan to or are currently participating in a clinical trial protocol for locoregional or systemic intervention, have been previously treated with MKIs for advanced disease or if they are in hospice (end-of-life) care.

### Treatments

The decision to treat with sorafenib or other MKIs (for e.g., lenvatinib) will be based on the investigator’s clinical judgment at study entry following confirmed disease progression. In this observational study, all drugs are prescribed in accordance with their marketing authorization based on current practice at the study sites.

### Endpoints

The primary endpoint is time to symptomatic progression (TTSP) defined as the time interval from the study entry to the date of first symptomatic progression. Patients with symptomatic progression, characterized as any sign and/or symptom or outcome caused by DTC, including symptoms due to bone metastasis; respiratory symptoms caused by metastatic lesion; central nervous system event due to brain metastasis; bleeding event; local discomfort or pain due to metastatic lesion; deterioration of general condition such as worsened ECOG performance status, fatigue and cachexia; reduced mobility due to metastatic lesion or death due to any cause, were considered to have met the primary endpoint.

The secondary endpoints are OS and PFS from the time of (1) study entry, (2) RAI refractory DTC diagnosis, (3) initiation of the first MKI, (4) initiation of each systemic treatment regimen and (5) initiation of sorafenib. Additional secondary endpoints are postprogression survival from the time of symptomatic progression, duration of and response to each systemic treatment regimen and dosing of sorafenib throughout the treatment period. Safety measurements assess AEs during treatment with sorafenib, including relation to treatment, seriousness, grade and outcome.

### Data collection

All study-related data will be collected by the investigator using case report forms via a Web-based online tool. Data collected in the first visit include initial diagnosis, history of disease, prior medication and diagnosis of RAI refractory disease. Follow-up visits occur during routine practice and are not defined in the study protocol. The final data collection is at patient’s death or at the end of study, whichever is earlier. Table 1 lists variables collected during the study. The variables required to determine the primary endpoint are treatment decision at the study entry and symptoms attributed to DTC. Disease progression will be assessed by the investigators. Safety assessment for patients treated with sorafenib includes all AEs, serious AEs and AEs leading to treatment discontinuation.

**Table 1:** Variables collected during the study.

**Variables**	**Initial visit**	**Follow-up visit(s)**	**Final visit**
Demography	X		
DTC history and classification (histology, stage/score at initial diagnosis and study entry)	X		
RAI history and DTC-refractory disease history	X		
Medical history	X		
ECOG performance status	X	X	X
Prior surgical and medical treatment for DTC	X		
Treatment decision on whether or not to start MKI at study entry	X		
Symptom of DTC (all patients)	X	X	X
Treatments for DTC, including MKIs, during the study	X	X	X
Laboratory parameters (per local standard of care)	X	X	X
Details on disease symptoms	X	X	X
Tumor assessment	X	X	X
Concomitant medication	X	X	X
Response assessment to treatment (per local standard of care)	X	X	X
Adverse events in patients treated with sorafenib		X	X
Hospitalization or unscheduled visits		X	

DTC, differentiated thyroid cancer; ECOG, eastern cooperative oncology group; MKI, multikinase inhibitor; RAI, radioactive iodine.

### Study size

Assuming a 1-sided α of 2.5%, a sample size of 700 patients is designed to have >80% power to detect 50% increase in median TTSP from study entry between the 2 cohorts with the assumption of median time of TTSP as 12 months from study entry in cohort 2 and expected loss to follow-up rate of 10%. Propensity score method used to balance baseline variables may reduce the number of patients who will be valid for primary analysis (Austin 2011). The sample size assumes 40–70% overlap of cohorts in the propensity score density function. Under these assumptions, 191–215 events will be required. An interim review assessing the assumed 50% propensity score density rate overlap will be conducted after 400 patients have been enrolled and may affect the planned sample size.

### Data analysis

All variables will be analyzed using descriptive statistics. Analysis will be performed for the total study population and, whenever reasonable, stratified by subgroups such as age, sex and baseline characteristics. Kaplan–Meier estimates will be calculated for time to event data. TTSP will be compared between the 2 study cohorts for statistical evaluation. A propensity score model, developed in an outcome-blinded manner by a third party, will be used to reduce confounding associated with nonrandomized studies. This approach balances measured baseline covariates and confounders between the 2 cohorts. The 2 secondary endpoints, OS and PFS from the time of study entry, will also be analyzed to compare cohorts 1 and 2. The remaining secondary endpoints will be analyzed to observe the survival of patients in the real-world clinical practice, and no comparison will be performed between the 2 cohorts.

For safety data, incidence of AEs during treatment with sorafenib will be summarized using the National Cancer Institute (NCI) Common Terminology Criteria for Adverse Events (CTCAE) and the latest Medical Dictionary for Regulatory Activities (MedDRA) version. Drug-related AEs, serious AEs and AEs leading to treatment discontinuation will also be summarized by NCI CTCAE grade.

## Discussion

RAI refractory DTC is a heterogeneous disease. This trial aims to determine whether initiating MKIs in asymptomatic patients at disease progression after RAI therapy is associated with increased time to symptomatic progression compared with not initiating MKIs. It focuses on asymptomatic patients since this population may not require immediate systemic treatment. A treatment algorithm devised by an expert panel provided a definition of RAI refractory disease that should be considered for systemic therapy (Schlumberger *et al.* 2014). These criteria (bulky tumors (>3 cm) or multiple tumors 1–2 cm in size that are rapidly progressing) are more stringent than the criteria used in our study, although the ultimate decision in this study depends on the investigator’s clinical judgment. Another multidisciplinary panel of experts also outlined patient characteristics that make them appropriate candidates for MKI therapy (Brose *et al.* 2012). The panel emphasized the importance of radiologic progression and also suggested patient’s choice and side effects of MKIs as additional factors to consider before the initiation of therapy. As RAI refractory DTC can follow an indolent course, physicians may adopt a watch and wait approach before initiating systemic therapy (Dadu & Cabanillas 2012). Results from the present study are expected to provide valuable information regarding the effect of the timing of treatment with MKIs on survival.

This study allows for observation of patients in a broad clinical setting, thus reflecting the real-world scenario. Medical decisions are based on mutual agreement between the patient and the physician. The prospective nature additionally allows for a more accurate measurement of exposure and clinical variables with the potential for collecting multiple outcomes as defined in the primary and secondary endpoints. The confounding typically present in nonrandomized studies will be minimized using a propensity score method, which will allow balancing covariate patterns between the study cohorts.

Selection bias is an inherent limitation of observational studies. Because this study aims to compare 2 nonrandomized cohorts of patients, all factors that could influence treatment assignment as well as outcome will have to be taken into account. To minimize the potential bias, eligible patients will be enrolled in a consecutive manner. An interim analysis has been included to assess the risk of low overlap in the propensity score density function between the 2 patient cohorts. However, even with an adequate overlap unmeasured confounders may still result in residual confounding. Therefore, HR estimates may not be completely free of bias, and the results should be interpreted with caution.

RIFTOS MKI is a large, global, noninterventional study designed to evaluate the use of MKIs in asymptomatic RAI refractory DTC patients. The study is expected to address the question of uncertainty regarding optimal timing of MKI initiation in asymptomatic patients by providing real-world data from routine clinical practice. Results from the study will enable the comparison of treatment practices and outcomes for patients with RAI refractory DTC and expand the knowledge base needed to optimize the use of MKIs.

## Declaration of interest

Marcia Brose has received consulting and grant support from Bayer and Eisai. Johannes Smit has received consulting and grant support from Bayer. Chia-Chi Lin has no relevant conflicts of interest to report. Fabian Pitoia has a consultant or speaker relationship with Bayer and Genzyme-Sanofi. Marc Fellous and Yoriko DeSanctis are employees of Bayer HealthCare Pharmaceuticals. Martin Schlumberger has a consultant or advisory relationship with AstraZeneca, Bayer, Eisai, and Genzyme-Sanofi; has received honoraria from AstraZeneca, Bayer, Eisai, Exelixis, Genzyme-Sanofi, and Sobi; and has received research funding from AstraZeneca, Bayer, Eisai, and Genzyme-Sanofi. Masayuki Tori has a consultant relationship with Bayer and Eisai; and has received honoraria from Bayer and Eisai. Iwao Sugitani has an advisory relationship with Bayer, Eisai and Genzyme-Sanofi; and has received grant support from Eisai.

## Funding

This work was supported by Bayer HealthCare Pharmaceuticals.

## Author contribution statement

All authors were involved in the study concept and design, and drafting and critical review of the manuscript. M B, J S, C L, F P, M S, M T and I S are the primary investigators for the study.
